# Interfacial, Electroviscous, and Nonlinear Dielectric Effects on Electrokinetics at Highly Charged Surfaces

**DOI:** 10.1021/acs.jpcb.0c11280

**Published:** 2021-05-03

**Authors:** Majid Rezaei, Bernhard G. Mitterwallner, Philip Loche, Yuki Uematsu, Roland R. Netz, Douwe Jan Bonthuis

**Affiliations:** † Fachbereich Physik, Freie Universität Berlin, 14195 Berlin, Germany; ‡ Department of Physics, Kyushu University, 819-0395 Fukuoka, Japan; § Institute of Theoretical and Computational Physics, Graz University of Technology, 8010 Graz, Austria

## Abstract

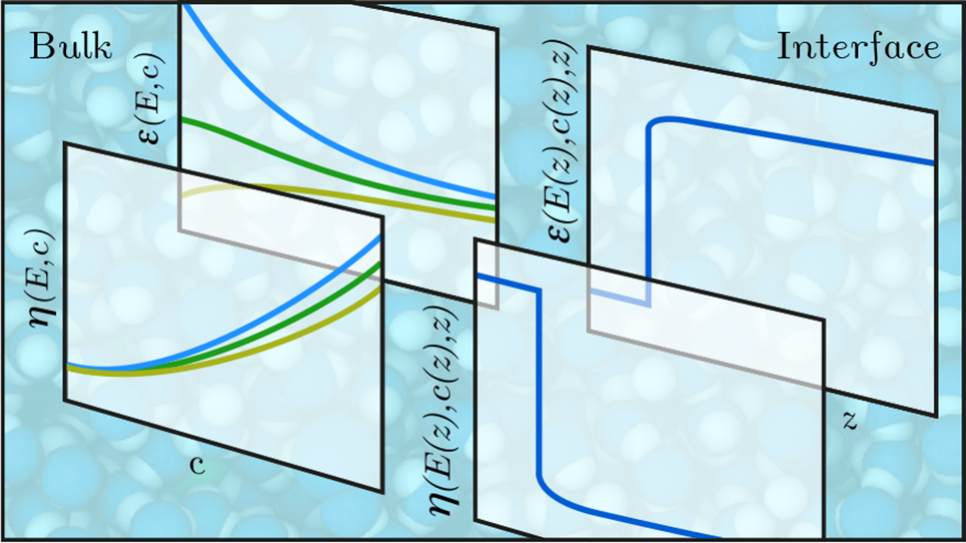

The dielectric constant and the viscosity of water at the interface of hydrophilic surfaces differ from their bulk values, and it has been proposed that the deviation is caused by the strong electric field and the high ion concentration in the interfacial layer. We calculate the dependence of the dielectric constant and the viscosity of bulk electrolytes on the electric field and the salt concentration. Incorporating the concentration and field-dependent dielectric constant and viscosity in the extended Poisson–Boltzmann and Stokes equations, we calculate the electro-osmotic mobility. We compare the results to literature experimental data and explicit molecular dynamics simulations of OH-terminated surfaces and show that it is necessary to additionally include the presence of a subnanometer wide interfacial water layer, the properties of which are drastically transformed by the sheer presence of the interface. We conclude that the origin of the anomalous behavior of aqueous interfacial layers cannot be found in electrostriction or electroviscous effects caused by the interfacial electric field and ion concentration. Instead, it is primarily caused by the intrinsic ordering and orientation of the interfacial water layer.

## Introduction

The molecular structure and dynamics of water are very sensitive to the presence of ions and macroscopic solutes.^1,2^ For example, with increasing ion concentration the static dielectric constant decreases^3^ and the viscosity increases or decreases depending on the ion type.^4−6^ In bulk solution, these effects are typically attributed to the presence of the strong electric field around the ions, causing saturation of the dielectric response, as well as to electroviscous effects (modified viscosity near charged solutes) and electrostriction (field-induced volume contraction).^7,8^ Also externally applied electric fields have an influence on the viscosity and the dielectric constant. As a function of the transverse electric field strength, the viscosity of various polar fluids is found to increase,^9^ but the effect is proportional to the conductivity of the fluid,^10^ raising the question of whether the electric field or the ion concentration dominates. On the basis of viscosity measurements between parallel electrodes, the coefficient of the electroviscous effect has been found to be positive with a negligible effect of the interfacial water layer.^11^ Molecular dynamics simulations of pure water in bulk complicate this picture further, indicating an enhanced viscosity component parallel to the electric field but a reduced viscosity in perpendicular direction for moderate electric fields.^12^ The measured dielectric constant of water decreases at strong electric fields.^13,14^

At charged interfaces, all of these effects coincide. Specifically, the interfacial dielectric constant and viscosity are expected to be different from their bulk values, because counterions accumulate at the interface, contributing to the electric field produced by the charged surface. In addition, the structure of the fluid around macroscopic solutes is transformed by the sheer presence of the interface,^15,16^ resulting in a finite-width interfacial layer where properties such as the dielectric constant and the viscosity are distinct from those in the bulk, even in the absence of surface charges, added salt and applied electric field.^17−20^ Which of these effects dominates the properties of charged interfaces in aqueous solution has remained an open question so far.

One of the properties of charged interfaces where these effects prominently manifest themselves is the electrokinetic mobility. Experiments at charged hydrophilic surfaces show that the mobility first increases with increasing surface charge density and then saturates.^21,22^ Early explanations of this effect have focused on electroviscous reduction of the electroosmotic flow, assuming continuum and bulk relations for the viscosity,^22,23^ which has long remained the accepted theory. Similarly, a salt-concentration-dependent dielectric constant has been used to model the disjoining pressure between charged plates, using bulk values for the dielectric decrement.^24^ More recently, a combination of molecular dynamics simulations and continuum theory has been used to show that the experimentally observed electrokinetic mobility can be accurately reproduced by assuming that an interfacial water layer is present with modified dielectric and viscous properties and that these properties remain equal to those at uncharged surfaces in pure water.^19,25,26^

In this paper, we investigate whether the effects of the electric field and the ion concentration on the water viscosity and dielectric constant that are found in bulk are sufficient to explain the dependence of the electrokinetic flow on the surface charge density or whether the effect of the interface on the water structure is necessary to reproduce the experimental results. We use atomistic molecular dynamics simulations to study the dielectric constant and the viscosity of bulk water as a function of the salt concentration and the electric field. Independently, we explicitly simulate the electroosmotic flow at a charged solid surface as a function of the surface charge density, showing good agreement with experimental data. Using the dielectric constant and the viscosity in the modified Stokes and Poisson–Boltzmann equations, we show that the bulklike dependence of the viscosity and the dielectric constant on the salt concentration and the electric field is insufficient to explain the observed electrokinetic flow. Instead, including an interfacial layer with a low dielectric constant and a high viscosity, caused by the radical transformation of the local fluid structure induced by the presence of the interface, produces good agreement with simulations and experiments. We conclude that the dominant contribution to the interfacial properties of charged solutes in water comes from the modified interfacial water layer, and that for an accurate model of the electrokinetic mobility, effects of the ion concentration and the electric field on the dielectric constant and the viscosity can be neglected.

## Experiments and Simulations Studying Interfacial Water Structure

The interfacial structure of water is characterized primarily by molecular orientation and layering. To study the structure of water and electrolytes at macroscopic interfaces, different surface-specific measurement techniques are used. The water orientation can be measured using second harmonic generation and sum-frequency generation. Sum-frequency spectroscopy shows that the hydrogen bond network at both hydrophobic and hydrophilic interfaces exhibits a stronger ordering than the one in bulk.^27^ Specifically, second harmonic generation reveals a strong orientation with the OH groups pointing toward the surface at quartz/water interfaces^28^ and silica/water interfaces,^29^ as well as alkane/water and PDMS/water interfaces.^30^ Naturally, this orientation is expected to have a strong effect on the dielectric constant at the interface,^31^ which is reproduced in molecular dynamics simulations of pure water at uncharged surfaces.^17,18^ Apart from orientation, fluids at a solid surface organize in layers, which has been observed in atomic force microscopy^32^ and shear force microscopy,^33^ and has also been found in molecular dynamics simulations of pure water.^17^ Although the layering is typically smeared out at soft and disordered surfaces, the interfacial density can still be different from the bulk, varying from depletion at hydrophobic surfaces^34^ to enhancement at hydrophilic ones.^35^ This local density, together with the hydrogen bond structure and the orientation is expected to have an effect on the local viscosity.^36^ Direct measurements of friction forces show that the interfacial viscosity differs from its bulk value indeed, with the deviation depending on the hydrophilicity of the surface: an enhanced interfacial viscosity is observed at hydrophilic silica and mica surfaces,^33,37^ but not at C and CH_3_-terminated surfaces.^37^ Also ultrasonic measurements show an enhanced interfacial viscosity at hydrophilic AlO_3_ surfaces,^35^ and a reduced viscosity at hydrophobic alkane/water interfaces.^38^ These effects have been reproduced in molecular dynamics simulations of pure water at a wall modeled by Lennard-Jones spheres.^26^ The consistent qualitative agreement between the experimental results and the atomistic simulations of pure water at uncharged surfaces suggests that the presence of the interface itself causes a structural change in the water, which manifests itself in the different values of the viscosity and the dielectric constant near the interface. On the basis of that hypothesis, the interfacial effects on the dielectric constant and the viscosity have been modeled using an effective interfacial layer.^17−19,25,26,39,40^ In order to quantitatively reproduce surface capacitances and electrokinetic measurements, however, some of the model’s parameters have to be fitted. Therefore, although the results discussed above show that the interfacial effects on the structure of pure water can cause the observed changes to viscosity and dielectric constant, they do not rule out any effects coming from surface charges or ions, which is the topic of the present work.

## Extended Electrokinetic Equations

### The Bulk Dielectric Constant

The dielectric constant quantifies the change of the polarization density with an applied electric field in the linear response regime, that is, when the applied field is small. At higher fields, the polarization density is a nonlinear function of the applied electric field, and thus the dielectric constant depends on the electric field strength as well. We calculate the differential dielectric constant of an electrolyte in bulk as a function of the applied electric field strength *E*_0_ and the salt concentration *c*_0_. The electric field is set by applying an external force *qE*_0_ in a chosen direction to all ionic and partial charges *q*. The differential dielectric constant in the bulk system is calculated using the fluctuation–dissipation relation^41^
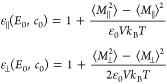
1with *M*_∥_ and *M*_⊥_ being the total polarization of the system in the directions parallel and perpendicular to the applied field, respectively, *k*_B_*T* being the thermal energy, ε_0_ being the vacuum permittivity, and *V* being the system volume. In eq 1, we have set the dielectric constant at high frequency to ε_*∞*_ = 1 because of the absence of atomic polarizability in our atomistic model.^42^

### The Poisson–Boltzmann Equation

At a charged surface, the polarization *M*(*z*), ion concentration *c*_±_(*z*), and electric field *E*(*z*) all depend on the distance *z* from the surface. For solving the Poisson–Boltzmann equation, a different definition of the dielectric constant is necessary. We start by writing the displacement field in terms of the polarization density *m*_∥_ in the direction of the electric field
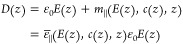
2which defines the dielectric difference profile ε̅_∥_(*E*(*z*), *c*(*z*), *z*), see Supporting Information. The separate *z* in the argument denotes an explicit dependence on *z*. Note that we assume that the dielectric response is local, depending on a single position *z*, which is valid for slowly varying displacement fields. In bulk simulations, the dielectric difference constant follows from eq 2 as
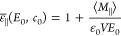
3

Note that the definitions of ε_⊥_, ε_∥_, and ε̅_∥_ coincide in the limit *E*_0_ → 0. On the mean-field level, the electrostatic potential ψ(*z*) obeys the one-dimensional Poisson–Boltzmann equation with a spatially varying local dielectric difference constant
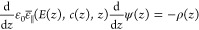
4with ρ(*z*) being the density of ionic charges and *E*(*z*) denoting the local electric field. Note that ε̅_∥_(*E*(*z*), *c*(*z*), *z*) denotes the dielectric difference tensor component parallel to the electric field, and thus perpendicular to the surface. We consider the case of monovalent ions with concentrations *c*_±_(*z*) for anions and cations, respectively. The charge density profile is thus given by
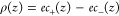
5where *e* denotes the elementary charge. For a given bulk salt concentration *c*_0_, the concentration profiles *c*_±_(*z*) are given by
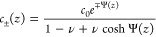
6

where Ψ(*z*) = *eψ*(*z*)/*k*_B_*T* denotes the reduced electric potential and  is the ionic packing parameter that accounts for steric repulsion between the ions. For a face-centered cubic (fcc) structure at maximum density, *d* denotes the effective steric diameter of both anions and cations. We set *d* = 0.3 nm, valid for typical monovalent ions.^43^ We consider the case of a single planar interface with surface charge density σ_0_. The potential is set to zero infinitely far away from the interface, that is, ψ(*z* → *∞*) = 0. Together with charge neutrality, ∫_0_^∞^d*z* ρ(*z*) = −σ_0_, this gives the second boundary condition
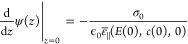
7

### The Interfacial Dielectric Profile

Apart from the electric field and the salt concentration, the dielectric difference constant in the Poisson–Boltzmann equation depends on the position *z*. We use two different models for the interfacial polarizability profile. First, we assume that the dependence on the local salt concentration and electric field is the same as in bulk everywhere. That means that the dependence of the interfacial dielectric constant on the local field *E*(*z*) and the local concentration *c*(*z*) is the same as the dependence of the bulk dielectric constant on the applied electric field *E*_0_ and the bulk concentration *c*_0_
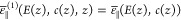
8

In this case, the anomalous electrokinetic behavior is caused by the strong electric field and high ion density in the interfacial layer only. Second, we assume that the interfacial layer behaves differently from the bulk due to the presence of the interface, whereas the rest of the fluid follows eq 8. For this scenario, we extend the box model that we have used previously,^40^
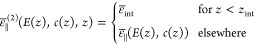
9

The quotient of the interfacial dielectric constant ε̅_int_ and the width of the interfacial layer *z*_int_ is extracted from molecular dynamics simulations of the dielectric profile.^17^ To achieve quantitative agreement with experimental data, the value of *z*_int_ is treated as a fit parameter, which simultaneously determines ε̅_int_.^40^ We will refer to the model of eq 9 as the “extended box model”. For a charged interface, the concentration profiles for anions and cations (*c*_+_ and *c*_–_, respectively) will be different. Since our bulk calculations of ε̅_∥_(*E*_0_,*c*_0_) only depend on the bulk salt concentration, we make the approximation that anions and cations have the same effect on the dielectric constant, defining the local ion concentration *c*(*z*) as the mean of *c*_+_(*z*) and *c*_–_(*z*)
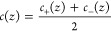
10

### The Bulk Viscosity

We estimate the components of the viscosity tensor in the directions parallel and perpendicular to the electric field of strength *E*_0_ in a bulk electrolyte of concentration *c*_0_ from the off-diagonal components of the Green–Kubo expression^44^
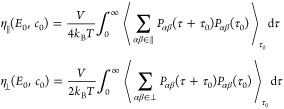
11with *V* being the volume of the simulation box and *P*_*αβ*_(τ) being the stress in the *αβ* plane as a function of time. Choosing *x* as the direction of the electric field, the sum in the first line of eq 11 is over *αβ* = {*xz*, *zx*, *xy*, *yx*} and the sum in the second line over *αβ* = {*yz*, *zy*}.

### The Stokes Equation

If an external electric field is applied parallel to a charged interface, an electroosmotic flow *u*(*z*) ensues which can be modeled by the one-dimensional Stokes equation. The viscosity at the interface exhibits a spatially varying profile.^25^ We assume that the viscosity near the interface depends on the salt concentration and on the local field from the surface and neglect the effect of the external electric field parallel to the surface, denoted *E*_ext_ to distinguish it from the external field *E*_0_ applied in the bulk systems. The external field is typically orders of magnitude smaller than the field due to the surface. The field due to the surface is perpendicular to the interface and thus also to the flow. We therefore have to consider the perpendicular component η_⊥_(*E*(*z*), *c*(*z*), *z*) of the viscosity:
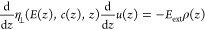
12

We use the Navier and charge-neutrality boundary conditions
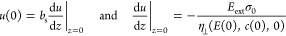
13where *b*_s_ denotes the slip length.^45^ With eq 13, eq 12 is solved for *u*(*z*) by integrating twice
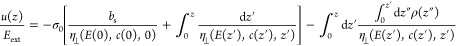
14

Together with the Poisson equation and its boundary conditions, eqs 4 and 7, eq 14 simplifies to
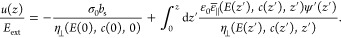
15

### The Interfacial Viscosity Profile

Like for the dielectric constant, we consider two models for the viscosity profile. In the first model, the electric field and concentration dependence is the same as in bulk everywhere,
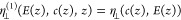
16

Second, we consider the case in which there is a box contribution to the viscosity profile^19,25^
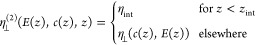
17

where *z*_int_ is the same as for the dielectric box model and η_int_ is the interfacial viscosity.

### Electrokinetics

We express the electrokinetic mobility in terms of the electrokinetic surface charge density. Combining the Stokes and the Poisson equations and using the viscosity η_w_ and the dielectric constant ε_w_ of bulk water in absence of an electric field for the entire fluid, the electrokinetic velocity can be expressed in terms of the zeta potential, which in this case corresponds to the electrostatic potential at the surface
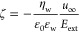
18

The velocity *u*_*∞*_ corresponds to the saturated value of the velocity far away from the surface. For planar channels, *u*_*∞*_ is defined as the velocity in the center of the channel if that velocity has saturated over a range of at least 1 nm. Assuming that the electrostatic potential at a charged surface is governed by the Poisson–Boltzmann equation, and again using ε_w_ for the dielectric constant, the surface potential can be expressed in terms of the corresponding surface charge density.^25^ This way, we can calculate the surface charge density corresponding to the electrokinetic velocity *u*_*∞*_ if the Stokes and Poisson–Boltzmann equations with η_w_ and ε_w_ were valid. This surface charge density is defined as the electrokinetic surface charge density σ_ek_^46^
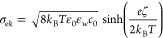
19

Note that although both the bulk dielectric constant and the bulk viscosity depend on the salt concentration, we adhere to the experimental convention of using ε_w_ and η_w_, respectively. In the absence of interfacial effects, electric field effects, concentration effects, and steric ion–ion interactions, the electrokinetic surface charge density coincides with the bare surface charge density, that is, ζ = ψ(0) and σ_ek_ = σ_0_.

## Simulations

Unless noted otherwise, all simulations are carried out using GROMACS versions 2016–2019, using a step size of 2 fs after energy minimization. We use the SPC/E water model^48^ and the GROMOS force field for the ions and the surfaces.^47^ The force field parameters are summarized in Table 1. The Lennard-Jones interactions are truncated at 0.9 nm without long-range dispersion correction, and we use the Particle Mesh Ewald summation in three dimensions with tinfoil boundary conditions for the long-range Coulomb interactions. The use of Ewald summation for the long-ranged electrostatics has been tested in two and three dimensions and found to be appropriate for the calculation of dielectric properties.^17,49^ The temperature is kept constant at 300 K using the v-rescale thermostat in all three dimensions. In the nonequilibrium simulations, we have compared the results to the results from simulations using the v-rescale thermostat only in the directions perpendicular to the flow,^50^ showing no discernible difference.

**Table 1:** Nonbonded Force Field Parameters Taken from GROMOS^47^^a^

	Na^+^	Cl^–^	OA	H	CH_2_	CH_3_	Si
σ (nm)	0.258	0.445	0.296	0	0.407	0.375	0.339
ϵ (kJ/mol)	0.0618	0.446	0.489	0	0.411	0.867	2.44
*q* (*e*)	1	–1	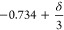	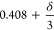		0	

a The CH_2_ and CH_3_ groups of the alcohols are modeled as united atoms. The first CH_2_ group after the hydroxyl carries a charge of *q* = 0.286+δ/3 *e* and the second and third CH_2_ groups carry *q* = 0.02 *e* each. The Si surface atoms in the slab simulations carry a partial charge of ±δ each. Geometric combination rules are used for interactions between dissimilar atoms.

### Bulk

Simulations of bulk water are performed in the presence of either an external electric field or with added NaCl or both. The simulations are performed in the *NPT* ensemble using the Berendsen barostat.

### Slab

As an alternative to applying an electric field to the bulk systems, one series of simulations is performed where a displacement field is applied by adding two oppositely charged plates, referred to as the “slab” system. The plates consist of four layers of silicon (Si) atoms arranged in an fcc-lattice with a lattice constant of *a* = 0.5431 nm, cut in the (111) direction. In the surface layer of one of the plates (directly adjacent to the fluid), the atoms carry a negative partial charge −δ, in the surface layer of the opposite plate, the atoms carry a positive partial charge δ, and all Si atoms which are not part of the surface layers are electrically neutral, leading to a surface charge density . The width of the channel, defined as the distance between the surface layers of Si atoms, is 4.84 nm. The number of water molecules is set at 2951 such that the initial pressure is zero. The dimensions of the simulation box are 4.66 × 4.50 × 22.0 nm and periodic boundary conditions are used in all directions. The long-ranged electrostatics are handled using two-dimensional P3M Ewald summation for the long-range electrostatic interactions, turning off electrostatic interactions between periodic boxes in the *z*-direction. These simulations are performed using LAMMPS.^51^

### Hydrophilic Surface

**Figure 1:**
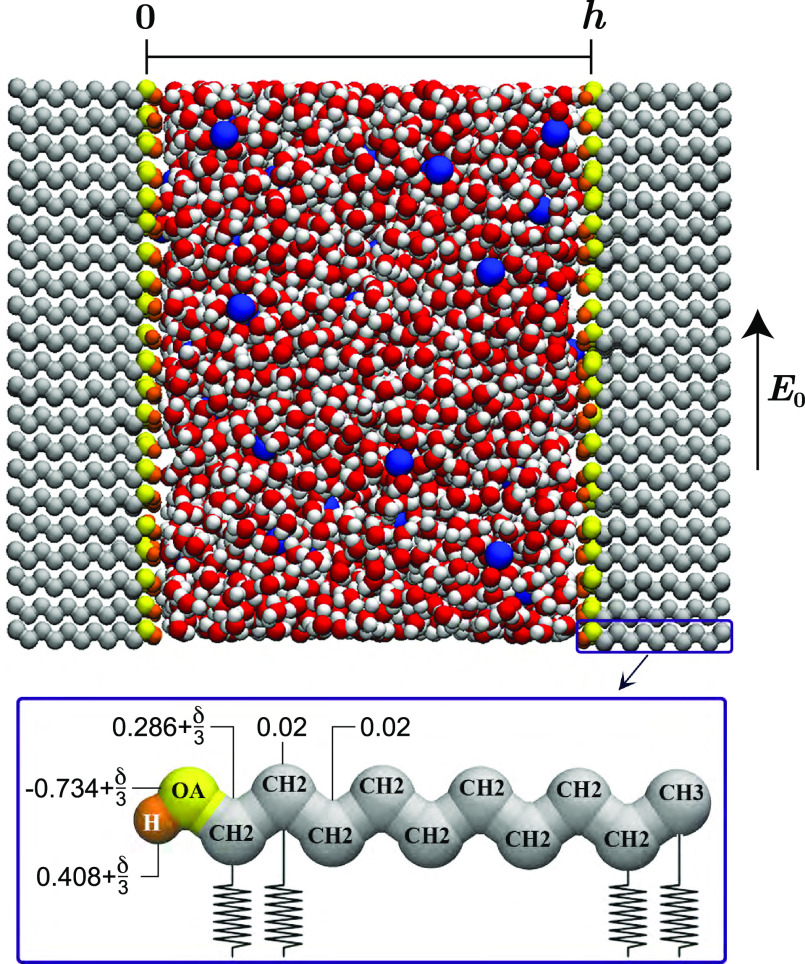
Snapshot of the simulation system with Na^+^ ions shown in blue and water in red and white. A magnified view of a decanol chain is shown at the bottom. Each chain carries a net charge δ. The first two and last two carbon atoms are restrained in a harmonic potential to keep the surface in place.

As a model hydrophilic surface, we use a layer of OH-terminated decanol molecules, see Figure 1. The molecules are restrained by the outer two carbon atoms on either end. The simulation system contains two layers of 100 decanol molecules each. Simulations are performed for two systems, for which the salt concentrations in the center of the box equal *c*_0_ = 25 ± 3 mM (system size set to 5.198 × 4.502 × 11.068 nm^3^) and *c*_0_ = 125 ± 10 mM (system size set to 5.198 × 4.502 × 6.95 nm^3^), respectively. The simulations are performed in the *NVT* ensemble, and the number of water molecules is determined such that the excess pressure vanishes. As a result, the number of water molecules and the concentration depend slightly on the surface charge density, giving rise to the error bars in the bulk concentrations. At zero surface charge density, the electrolyte contains either 2 NaCl pairs and 6583 water molecules (∼25 mM) or 5 NaCl pairs and 3277 water molecules (∼125 mM). A finite surface charge density is set by distributing an integer number of unit charges evenly over the O, H and outer C atoms of the decanol chains. To neutralize the charge, the number of Na^+^ ions equals the sum of the number of Cl^–^ ions and the total surface charge.

### Nonequilibrium Electrokinetics

The applied electric field parallel to the decanol surfaces equals *E*_0_ = 0.3 V/nm. The systems are simulated for 70 ns, of which the final 40 ns are used for the calculation of the velocities. We set the velocity of the center of mass to zero and report the velocity differences between the surface and the fluid in the region where the velocity has saturated. At a salt concentration of 25 mM, we have tested the effect of the surface ordering and flexibility by freezing the surface atoms in one series of simulations.

## Results and Discussion

### Bulk Dielectric Constant

**Figure 2:**
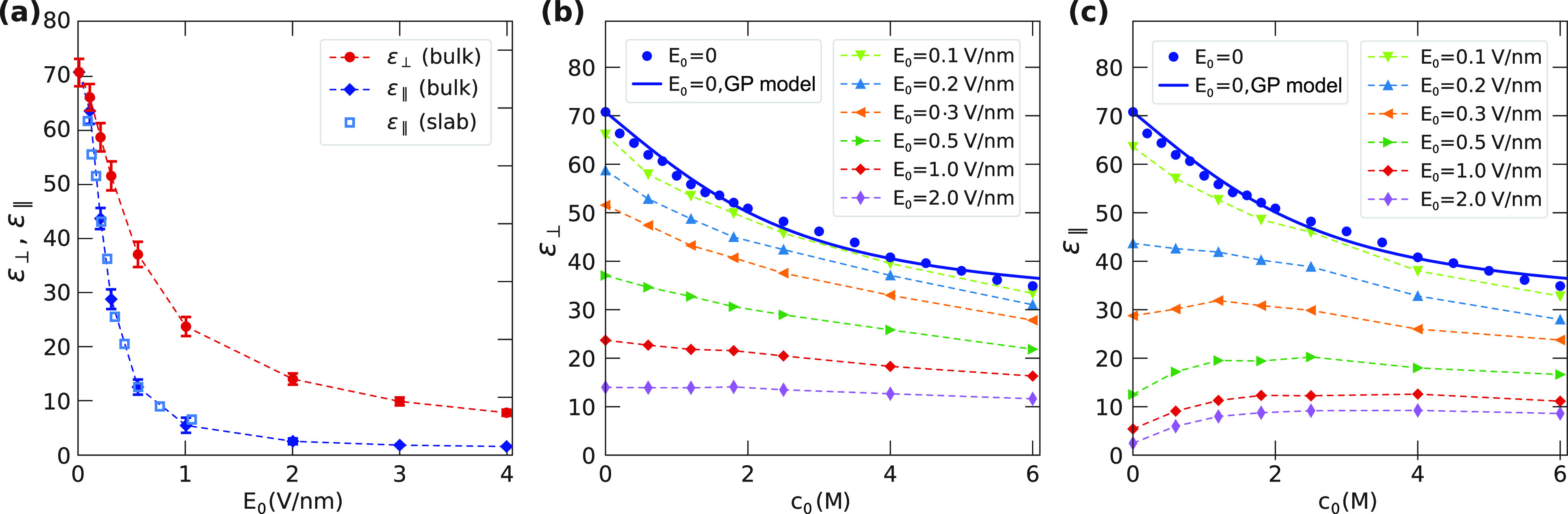
(a) The bulk differential dielectric constant at *c*_0_ = 0 in the directions perpendicular and parallel to the electric field, calculated from bulksimulations using the fluctuation eqs 1 (solid symbols) and from slab simulations (open symbols). (b,c) The perpendicular and parallel components of the dielectric constant as a function of the salt concentration for different values of the electric field calculated from eqs 1. At *E*_0_ = 0 V/nm, the concentration dependence of ε_⊥_(0, *c*_0_) = ε_∥_(0, *c*_0_) is fitted with the Gavish–Promislow (GP) eq 20 (solid line). The broken lines serve as guides to the eye. Error bars in panels (b,c), which are similar in magnitude compared to the ones in panel (a), have been omitted for clarity.

The parallel and perpendicular components of the dielectric constant of bulk water as a function of the applied electric field in the absence of salt ions, calculated from the polarization fluctuations using eq 1, are shown in Figure 2a. In the absence of both electric field and salt ions, we obtain a bulk water dielectric constant of ε_w_ = 71 ± 2, in agreement with the literature value of 71 for the SPC/E water model.^18^ The bulk dielectric constant is very sensitive to the applied electric field. In particular, the component parallel to the applied field exhibits a very steep decline, dropping to less than 10% of ε_w_ at *E*_0_ = 1 V/nm. To verify the results, ε_∥_ is also calculated from a series of simulations in the slab geometry, where different constant fields *D* are applied by means of two oppositely charged plates with surface charge density ±σ. The corresponding electric fields *E*(*z*) are calculated from ε_0_*E*(*z*) = *D* – *m*_∥_(*z*), where the usual contribution from the periodic images vanishes because of the two-dimensional Ewald summation used in the simulations. The electric-field dependent differential dielectric constant is calculated from the change in electric field in the center of the box (*z* = *h*/2) in response to a change in the applied displacement field, ε_∥_(*E*, 0) = Δ*D*/(ε_0_Δ*E*(*h*/2)). The changes Δ*D* and Δ*E*(*h*/2) are calculated from two subsequent simulations at different surface charge densities, and the calculated ε_∥_(*E*, 0) is assigned to the average electric field *E*(*h*/2) in these two simulations. The results from the fluctuation–dissipation eq 1 (solid symbols, denoted bulk) and the results from the slab system (open symbols, denoted slab) are in excellent agreement, as shown in Figure 2a.

At finite salt concentrations, we calculate the dielectric constant from the fluctuations of the water polarization only, neglecting the ion–water and ion–ion correlations. To check that this procedure is justified, we also calculate the complete dielectric spectrum from the fluctuations of the current density, see Appendix A. The contribution of the ion–ion and ion–water correlations is less than 3%, in agreement with previous results,^52^ validating our approach. At zero electric field, the dielectric constant decreases as a function of the salt concentration, as shown in Figure 2b,c. The Gavish–Promislov model has been used to describe the dependence of the dielectric constant on the salt concentration *c*_0_,^53^
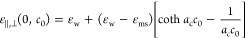
20

where ε_ms_ is the limiting value of the dielectric constant for very high salt concentrations and *a*_c_ is a fit parameter which is related to the excess polarizability α of the ions via *a*_c_ = 3α/(ε_w_ – ε_ms_). The solid curves in Figure 2b,c show that eq 20 provides an excellent fit to the simulation data. We obtain
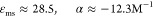


Comparing to experimental data, the values of ε_ms_ and α are in good agreement with the values reported for NaCl (ε_ms_ = 27.9, α = −11.59 M^–1^),^54^ despite the fact that we use an ion force field which has not been optimized to reproduce the electrolyte thermodynamics. At high electric fields, the dependence of the dielectric constant on the salt concentration becomes a lot less pronounced. This can be understood by realizing that due to the decreasing dielectric constant as a function of the external electric field, the negative excess polarizability due to the perturbation of the water surrounding the ions vanishes at some point, after which it turns positive. That means that above a threshold *E*_0_, the ions disorder the field-aligned water and the dielectric constant increases as a function of the salt concentration. As can be seen in Figure 2c, for ε_∥_(*E*_0_, *c*_0_), this transition occurs between *E*_0_ = 0.3 V/nm and *E*_0_ = 0.5 V/nm, when the dielectric constant of the environment decreases to a value below ε_ms_ (see Figure 2a). For ε_⊥_(*E*_0_, *c*_0_), even though the salt concentration dependence is greatly diminished at *E*_0_ ≈ 2 V/nm, the transition to positive α is not observed, which can be understood by considering the higher value of ε_⊥_(*E*_0_, 0) over the full range of *E*_0_.

### Bulk Dielectric Difference Constant

We calculate the dielectric difference constant from the bulk simulations using eq 3. We have verified that the result is equivalent to integrating ε_∥_(*E*_0_, 0) over *E*_0_ and dividing by *E*_0_.

**Figure 3:**
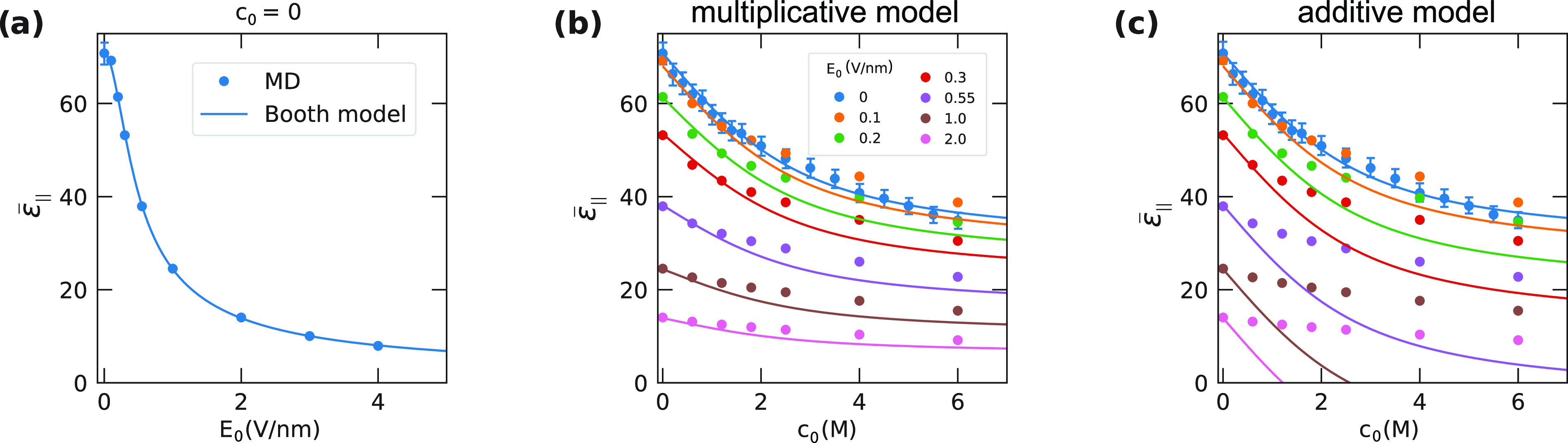
(a) The bulk dielectric difference constant ε̅_∥_(*E*_0_,0) obtained from MD simulations at zero salt concentration (symbols) fitted with the Booth model of eq 21 (solid line). (b,c) Bulk dielectric difference constant ε̅_∥_(*E*_0_,*c*_0_) as a function of salt concentration for different values of the external electric field (symbols). The solid lines show the Gavish–Promislov model of eq 20 and the Booth model of eq 21, combined according to (b) the multiplicative model of eq 22 and (c) the additive model of eq 23. The error bars for ε̅_∥_ at nonzero electric field are smaller than the symbol size.

At zero salt concentration, the bulk dielectric difference constant ε̅_∥_(*E*_0_,0) is shown in Figure 3a as a function of the applied electric field. If many-body effects are neglected, the polarization *M*_∥_ is described by the nonlinear dielectric response of a simple dipole, as put forward by Booth.^55^ Using eq 3 leads to the following expression for the dielectric difference constant
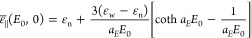
21with ε_n_ and *a*_E_ being fit parameters. Equation 21 perfectly fits the data obtained from the MD simulations, see Figure 3a. We obtain
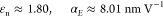


As a function of the salt concentration, the simulated dielectric difference constants at different electric field strengths are shown as symbols in Figure 3b,c. The blue symbols depict the dielectric difference constants at zero electric field, ε̅_∥_(0,*c*_0_), which are fitted well by eq 20 because ε_∥_(0,*c*_0_) = ε_⊥_(0,*c*_0_) = ε̅_∥_(0,*c*_0_).

Having successfully obtained the fit functions of the dielectric difference constant in the limits of vanishing electric field and vanishing salt concentration, we now consider two different expressions for the case in which both salt concentration and external field are nonzero. First, we make a multiplicative ansatz
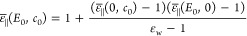
22

Our ansatz ensures that ε̅_∥_(0,0) = ε_w_ and ε̅_∥_(*c*_0_ → ∞,*E*_0_ → ∞) ≥ 1. Alternatively, we consider a model where the combined effect of salt and field is additive
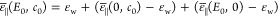
23

Comparing these two models with simulation data singles out the multiplicative ansatz of eq 22 as the better model, see Figure 3b,c. This result agrees with the derivation presented by Gavish and Promislow, where the external field and the field due to the ions are considered to be additive, leading to a multiplicative expression for the dielectric response.^53^

### Bulk Viscosity

**Figure 4:**
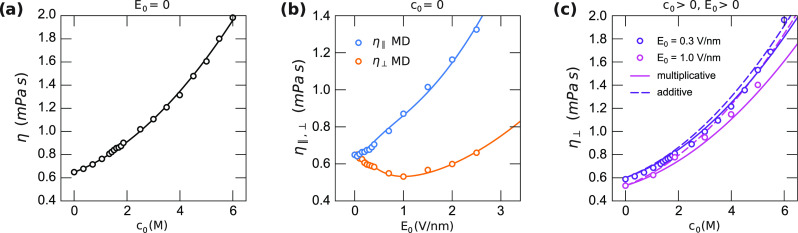
Bulk viscosity obtained from MD simulations (symbols) as a function of the salt concentration at zero external field (a) and as a function of electric field at zero salt concentration (b). Solid lines show the fits of eq 24 and 25, respectively. (c) The perpendicular component η_⊥_ of the bulk viscosity in the presence of both salt and external electric field. Solid lines show the multiplicative ansatz (first line of eq 26) and dashed lines show the additive ansatz (second line of eq 26). The error bars are of the order of the symbol size.

We extract the bulk viscosity from simulation data via the Green–Kubo relation given in eq 11. The bulk viscosity calculated for pure SPC/E water in the absence of an electric field is η_w_ = η_⊥_(0, 0) = η_∥_(0, 0) = 0.648 ± 0.002 mPa s, which is in good agreement with the values reported in the literature^56,57^ but significantly lower than the experimental bulk viscosity of water, 0.798 mPa s at a temperature of 303 K.^58^ As for the dielectric constant, we calculate the viscosity also in the presence of salt and at finite electric field, see Figure 4. For the dependence of the viscosity on the salt concentration, we choose a second degree polynomial as a phenomenological formula which perfectly fits the data, see Figure 4a
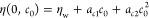
24

Note that the asymptotic of the concentration dependence of the viscosity at low *c*_0_ is in fact proportional to ,^6^ which becomes significant at low concentrations (*c*_0_ < 0.5 M). At the high concentrations we are treating here, however, we include the quadratic term instead. For the fit parameters, we obtain *a*_*c*1_ = 0.0777 mPa s/M and *a*_*c*2_ = 0.0223 mPa s/M^2^. The components of the viscosity perpendicular and parallel to the electric field are shown in Figure 4b as a function of electric field at *c*_0_ = 0. Whereas the parallel component η_∥_(*E*_0_, 0) increases with *E*_0_, the perpendicular component η_⊥_(*E*_0_, 0) first decreases. In order to use the viscosity in the Stokes equation, we construct a heuristic fit function to interpolate the viscosity. At high electric field, the viscosity increases quadratically, and for symmetry reasons the viscosity needs to be a function of even powers of the field *E*_0_. We make a simple empirical ansatz for η_⊥,∥_(*E*_0_, 0) which is consistent with these requirements
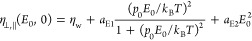
25

where *a*_E1_, *a*_E2_ are fit parameters and *p*_0_ is the dipole moment of a single water molecule for which we use the value for SPC/E water, *p*_0_ = 0.049*e* nm. The first part of eq 25 can be expanded as an infinite sum over all even powers of *E*_0_ with a single prefactor. The final term quantifies the increase of the viscosity at high electric field. The MD data for both η_∥_ and η_⊥_ can be described well with eq 25, see Figure 4b. We obtain *a*_E1_ = 0.160 mPa s and *a*_E2_ = 0.0868 mPa s/(V/nm)^2^ for the parallel case and *a*_E1_ = −0.190 mPa s and *a*_E2_ = 0.0319 mPa s/(V/nm)^2^ for the perpendicular case.

Like for the dielectric difference constant, we attempt both a multiplicative and an additive ansatz to fit the viscosity at finite field and concentration
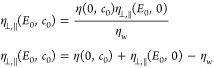
26

Figure 4c shows a comparison of the two models of eq 26 for η_⊥_(*E*_0_, *c*_0_). As the multiplicative ansatz performs slightly better, we choose the multiplicative ansatz for further analysis.

### Electrokinetics

**Figure 5:**
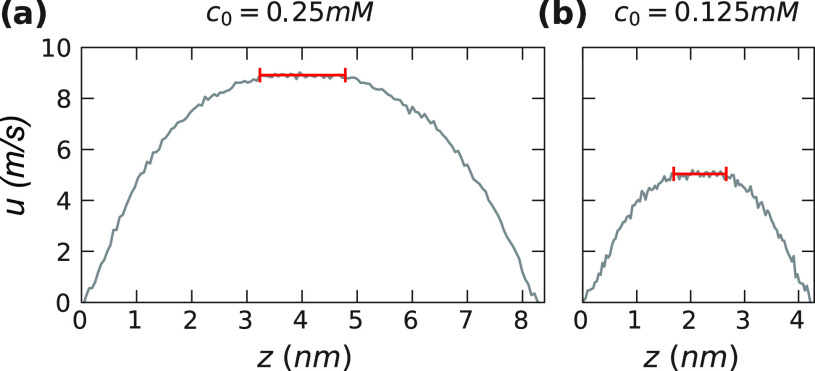
Average velocity of the fluid (water and ions) with respect to the hydrophilic surface with charge density σ_0_ = 0.1 *e*/nm^2^ at bulk concentrations of (a) *c*_0_ = 25 mM and (b) *c*_0_ = 125 mM. The range used to calculate *u*_*∞*_ is indicated in red.

**Figure 6:**
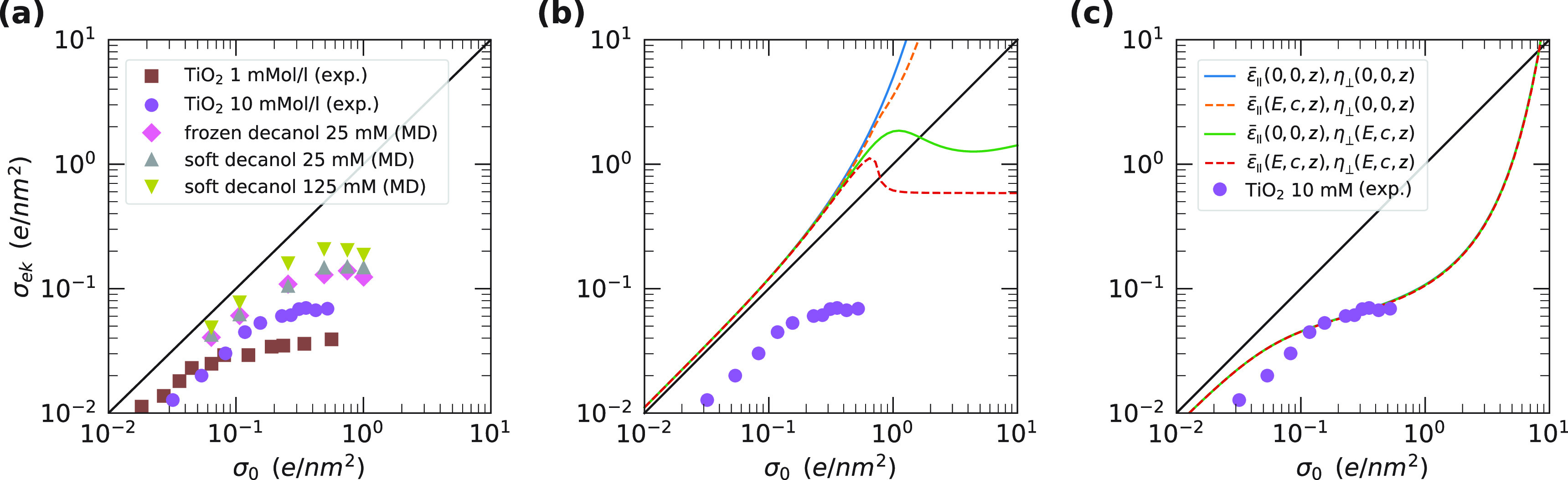
Electrokinetic surface charge density σ_ek_ as a function of bare surface charge density σ_0_. (a) Results of the electrokinetic simulations of a 25 and a 125 mM aqueous solution of NaCl in contact with a hydrophilic surface consisting of decanol chains. At 25 mM, we test the effect of the flexibility of the surface (diamonds and upward triangles). The error bars for the simulation data are of the order of the symbol size. The experimental data of a TiO_2_ surface in contact with a KNO_3_ solution are shown for comparison.^59^ (b) Results of the Stokes–Poisson–Boltzmann calculation using the bulk relations ε̅_∥_^(1)^(*E*(*z*),*c*(*z*)) (eq 8) and η_⊥_^(1)^(*E*(*z*),*c*(*z*)) (eq 16). (c) Results for the extended box model (eqs 9 and 17), for all combinations of field and concentration dependent ε̅_∥_^(2)^(*E*(*z*),*c*(*z*),*z*) and η_⊥_^(2)^(*E*(*z*),*c*(*z*),*z*) (note that all lines overlap).

In Figure 5, we show the velocity profile for *c*_0_ = 25 mM and *c*_0_ = 125 mM, obtained in charged channels of different height *h*, see the Supporting Information for details. We average the velocity profile in the center of the channel (red bars in Figure 5), where it reaches a constant value over a range of at least one nanometer. Figure 6a shows the electrokinetic surface charge density, defined in eq 19, obtained from explicit molecular dynamics simulations at the hydrophilic surface, together with the experimental data of TiO_2_ colloids at different salt concentrations.^59^ Clearly, the trend of the experimental data is well reproduced with the electrokinetic surface charge density increasing sublinearly with increasing bare surface charge density. The simulated saturation value increases with increasing salt concentration in line with the experimental trend. Note that we used 25 mM as the lowest concentration in the simulations, because the lower values of *c*_0_ necessary for a direct comparison with the experiments would require significantly larger simulation box sizes.

Now we test the ability of the different scenarios to reproduce the experimental electrokinetic surface charge density as a function of the bare surface charge density by solving the Stokes and Poisson–Boltzmann equations with the models for the dielectric constant given in eqs 8 and 9 and the viscosity given in eqs 16 and 17. First, we show σ_ek_ using the bulk dielectric difference constant ε̅_∥_(*E* = 0, *c* = 0) and bulk viscosity η_⊥_(*E* = 0, *c* = 0) at *c*_0_ = 10 mM in Figure 6b (blue solid line). The electrokinetic surface charge density exceeds σ_0_ over the entire range of σ_0_, which is caused by the steric repulsion between the ions included in eq 6. Including the bulklike dependence of the dielectric difference constant on the local ion concentration and electric field, modeled by ε̅_∥_^(1)^(*E*(*z*),*c*(*z*)) according to eq 8, only has a minor effect (orange broken line in Figure 6b). Including the bulklike dependence of the viscosity on the local ion concentration and electric field, modeled by η_⊥_^(1)^(*E*(*z*), *c*(*z*)) according to eq 16, does give rise to a saturation of σ_ek_, but only for σ_0_ > 1 *e*/nm^2^, after an initial superlinear increase (green solid and red broken lines in Figure 6b). Clearly, none of the curves in Figure 6b reproduce the experimental data, showing that the properties of the interfacial layer at charged surfaces cannot be reproduced by the bulklike dependence of the viscosity and dielectric constant on the salt concentration and electric field.

In Figure 6c, we use the extended box model of eqs 9 and 17 at 10 mM, showing good agreement with the experimental data. We use the interfacial parameters from ref (40), see Table 2. The values of η_int_/η_w_ and ε̅_int_/*z*_int_ have been obtained from molecular dynamics simulations of the interfacial viscosity and the dielectric profile of pure water at OH-terminated surfaces. To find the value of *z*_int_, the assumption has been made that the width of the interfacial layer is the same for the viscous and dielectric properties. In fact, including the dependence on ion concentration and dielectric constant has a negligible effect on the electrokinetic surface charge density (the curves in Figure 6c overlap) showing that the behavior is dominated by the structure of the interfacial water layer.

**Table 2:** Interfacial Parameters for a TiO_2_ Surface in Contact with a 1 mM KNO_3_ Solution Taken from Ref (40)

*d* (nm)	*z*_int_ (nm)	ε̅_int_	η_int_/η_w_	*b*_s_ (nm)
0.3	0.44	4.4	3.7	–0.32

**Figure 7:**
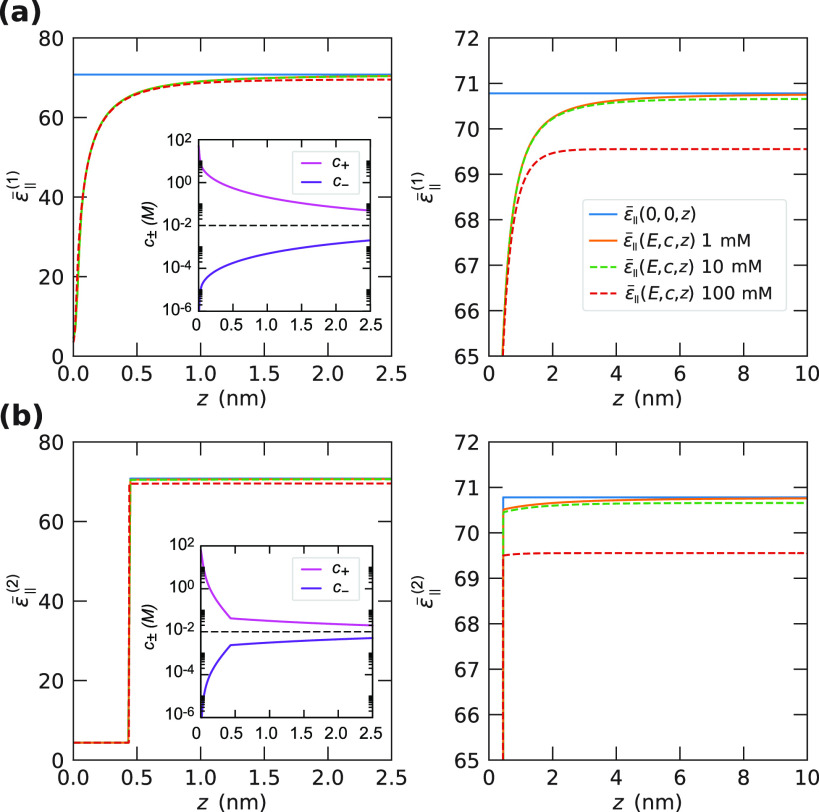
Dielectric difference profiles obtained from the Poisson–Boltzmann solution at a surface charge density of σ_0_ = 1*e* nm^–2^. The box parameters are the same as in Table 2. (a) The dielectric difference constant in the absence of the interfacial box, ε̅_∥_^(1)^(*E*(*z*), *c*(*z*)), eq 8. (b) The extended box model, ε̅_∥_^(2)^(*E*(*z*), *c*(*z*), *z*), eq 9. The case ε̅_∥_(*E* = 0, *c* = 0, *z*) corresponds to a constant dielectric constant (a) or the standard dielectric box model (b),^40^ respectively. The ion density profiles *c*_±_(*z*) corresponding to *c*_0_ = 10 mM are shown in the insets. The panels on the right-hand side are magnifications of the panels on the left-hand side. Note that the dielectric profiles for ε̅_∥_(*E*(*z*), *c*(*z*), *z*) do not saturate to ε_w_ due to the nonvanishing salt concentration in bulk.

**Figure 8:**
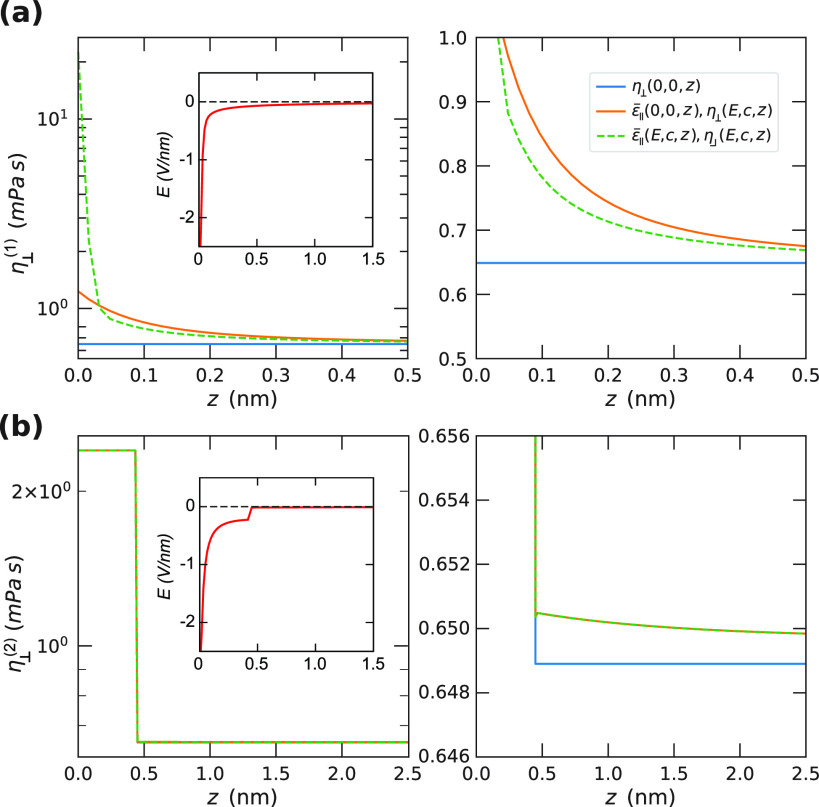
Viscosity profiles obtained from the Poisson–Boltzmann solution at a surface charge density of σ_0_ = 1*e* nm^–2^ and a bulk salt concentration of *c*_0_ = 10 mM. The box parameters are the same as in Table 2. (a) The viscosity profile η_⊥_^(1)^(*E*(*z*), *c*(*z*)), eq 16, in the absence of the interfacial box. (b) The extended box model, η_⊥_^(2)^(*E*(*z*), *c*(*z*), *z*), eq 16. The case η(*E* = 0, *c* = 0, *z*) corresponds to a constant viscosity (a) or the standard box model (b), respectively, independent of the dielectric constant. The electric field profiles when using (a) ε̅_∥_^(1)^(*E*(*z*), *c*(*z*)) and (b) ε̅_∥_^(2)^(*E*(*z*), *c*(*z*), *z*) are shown in the insets. The panels on the right-hand side are magnifications of the panels on the left-hand side.

Why does the dependence of the viscosity and dielectric constant on the local electric field and salt concentration have a negligible effect on the electrokinetic mobility? To see why this is the case, Figure 7 shows a comparison of the resulting dielectric difference profiles ε̅_∥_(*E*(*z*), *c*(*z*), *z*) for various salt concentrations at a surface charge density of σ_0_ = 1*e* nm^–2^. Without interfacial box model, the effect of the electric field and the salt concentration is substantial, strongly reducing the dielectric constant close to the interface, see Figure 7a. However, we find that for the extended box model, the profiles are dominated by the box contribution, while the effect of the salt and field dependence is much smaller in comparison because the electric field and the salt concentration quickly decrease beyond the interfacial layer, see Figure 7b. Similarly, the resulting viscosity profiles η_⊥_(*E*(*z*), *c*(*z*), *z*) are shown in Figure 8, panel a for the bulklike electric field and concentration dependence of eq 16 and panel b for the extended box model of eq 17 at a surface charge density of σ_0_ = 1*e* nm^–2^ and a bulk salt concentration of *c*_0_ = 10 mM. Whereas in Figure 8a, the electric field and the salt do lead to an increased viscosity close to the surface, the contribution from the box model shown in Figure 8b is significantly stronger over a much longer range. The corresponding ionic densities and electric field at a concentration of 10 mM and σ_0_ = 1 *e*/nm^2^ are shown in the insets of Figures 7 and 8. On the basis of Figure 6, it is clear that the effect of the surface on the interfacial dielectric constant and the interfacial viscosity cannot be modeled by the profiles shown in Figure 7a and Figure 8a. Instead, the presence of the interface has such a drastic effect on the water structure, modeled by the box contributions in Figure 7b and Figure 8b, that the additional effects of ions and electric field can be safely ignored. This explains why our previously used model,^19,25^ using only the effect of the interfacial water layer on the dielectric and viscosity profiles, provides good agreement with the available experimental data.

## Conclusions

We have studied the dependence of the dielectric constant and the viscosity in bulk water and at interfaces as a function of the applied electric field and the salt concentration. For the bulk dielectric constant, both the components parallel and perpendicular to the applied electric electric field decrease as a function of the field strength, in agreement with the Booth model.^55^ The decrease is significantly steeper for the parallel component. At low electric field, the dielectric constant also decreases with increasing salt concentration, but at high electric field, the dielectric constant parallel to the electric field increases with the salt concentration. The combined effects of concentration and electric field can be modeled using the Booth model for the field dependence and the Gavish–Promislow model for the salt concentration dependence in a multiplicative ansatz.

The bulk viscosity increases with increasing salt concentration, which can be modeled using a second order polynomial. Parallel to an applied electric field, the viscosity also increases with electric field strength, but in perpendicular direction the viscosity first decreases. This means that the electroviscous effect of the perpendicular viscosity starts only at a much higher field strength.

Fitting the dielectric constant as a function of salt concentration with the Gavish–Promislow model, our simulations achieve quantitative agreement with experimental values for the effective ionic polarizability and the high-salt limit of the dielectric constant. In general, however, these values are expected to be sensitive to the model used for the water and the ions. Similarly, the bulk dielectric constant of pure water is slightly underestimated by the SPC/E water model, and the simulated viscosity of pure water is even significantly lower than the experimental value. Therefore, a careful optimization of the water and ion force fields would be required before rigorous quantitative conclusions can be drawn.

The dependencies of the dielectric constant and the viscosity on the field and ion density also affect the interfacial layer at charged interfaces. However, using the bulklike functional form for the dielectric constant and the viscosity in the Poisson–Boltzmann and Stokes equations, the experimentally measured electrokinetic surface charge density cannot be reproduced. Instead, we use an extended box model, where in addition to the electric field and ion density dependence, the dielectric constant and the viscosity exhibit an interfacial layer where the presence of the surface drastically changes the dielectric constant and the viscosity of the interfacial water layer. Using this extended box model, the experimental data are well reproduced. In fact, the dependence of the dielectric constant and the viscosity on the electric field and the ion concentration turns out to be negligible in comparison to the effect of the interfacial water layer.

Using explicit MD simulations, which incorporate both the effects of the interfacial water layer and the ions and electric field, we reproduce the saturation observed in the experimental electrokinetic surface charge density as a function of the bare surface charge density. The bulk concentrations used in the simulations are slightly higher than those used in experiments, complicating a direct quantitative comparison, but the trend of increasing electrokinetic surface charge density with increasing salt concentration is well reproduced.

Our results show that at moderate surface charge densities and bulk salt concentrations, electrokinetics can be modeled using the interfacial properties of the pure water interface, and the additional effects of the high ionic density and strong electric fields on the interfacial dielectric constant and viscosity can be ignored.

## Figures and Tables

**Figure 9:**
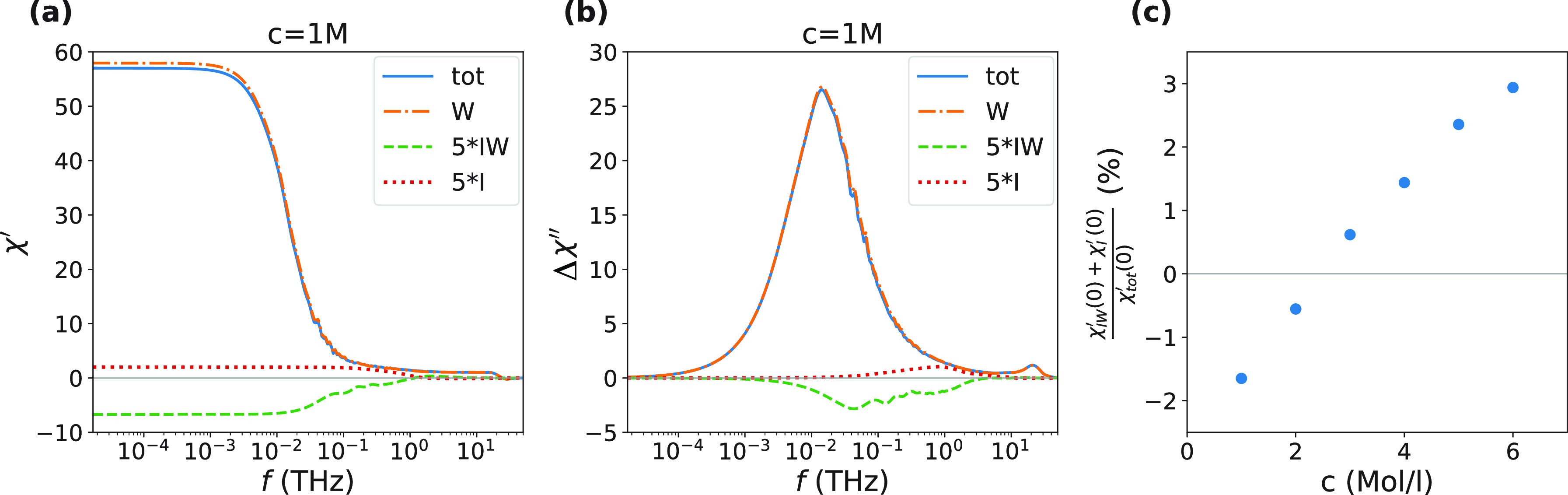
(a) Real and (b) imaginary parts of the dielectric spectrum of NaCl in water at a concentration of *c*_0_ = 1 M. The spectrum has been split into contributions from the water–water (W), ion–water (IW), and ion–ion (I) correlations. (c) The relative contribution of the ion–ion and the ion–water correlations.

## Appendix A: Dielectric Spectrum

We calculate the dielectric spectrum of a NaCl solution, split into the water–water, ion–water and ion–ion contributions according to the method explained in ref (52). Briefly, the frequency dependent susceptibility χ(*f*) = ε(*f*) – 1 = χ′(*f*) – *iχ*″(*f*) is given by the fluctuation–dissipation relation

27

with *V* being the system volume and *Ṁ*(*t*) being the time derivative of the polarization. The imaginary part of the susceptibility, χ″(*f*), diverges at low frequency due to the ionic DC conductivity. Therefore, we report the DC-conductivity-corrected susceptibility Δχ(*f*) = χ(*f*) + *is*_0_/(2*πf*), where *s*_0_ is the static ionic conductivity. By splitting the polarization into contributions from the water, *M*_W_(*t*), and contributions from the ions, *M*_I_(*t*), the spectrum is split according to Δχ(*f*) = χ_W_(*f*) + χ_IW_(*f*) + Δχ_I_(*f*). The parts χ_W_(*f*), χ_IW_(*f*), and Δχ_I_(*f*) correspond to the contributions from the water–water, ion–water, and ion–ion correlations, respectively. The latter two are conveniently calculated via correlations of the ionic current *J*_I_(*t*) = *Ṁ*_I_(*t*) with the water polarization *M*_W_(*t*), and with itself, respectively. The spectrum at a concentration of 0.6 M (zero applied electric field) is shown in Figure 9a as a function of the frequency *f*, showing that the water–water contribution dominates the spectrum over the entire frequency range. Figure 9b shows the relative contribution of the ion–ion and ion–water terms at *f* = 0 for different salt concentrations. The results show that ignoring the ion–water and ion–ion correlations when calculating ε_∥,⊥_(*E*_0_, *c*_0_) in this system leads to an error of at most 3*%*.

## Supporting Information Available

Calculation of the local electric field, definition of the dielectric constant, and details of the velocity calculations (PDF)
