# Supplementary material for: Interfacial, Electroviscous, and Nonlinear Dielectric Effects on Electrokinetics at Highly Charged Surfaces
Source: J Phys Chem B. 2021 May 3;125(18):4767–78. doi: 10.1021/acs.jpcb.0c11280 (PMC8154604; doi:10.1021/acs.jpcb.0c11280)
Supplement: Supplementary file 1 — jp0c11280_si_001.pdf [file jp0c11280_si_001.pdf]

# Supplementary information for "Interfacial, electroviscous, and nonlinear dielectric effects on electrokinetics at highly charged surfaces"

Majid Rezaei,[1,*] Bernhard G. Mitterwallner,[1,*] Philip Loche,[1]
Yuki Uematsu,[2] Roland R. Netz,[1] and Douwe Jan Bonthuis[3,†]
[1]*Fachbereich Physik, Freie Universität Berlin, 14195 Berlin, Germany*
[2]*Department of Physics, Kyushu University, 819-0395 Fukuoka, Japan*
[3]*Institute of Theoretical and Computational Physics, Graz University of Technology, 8010 Graz, Austria*

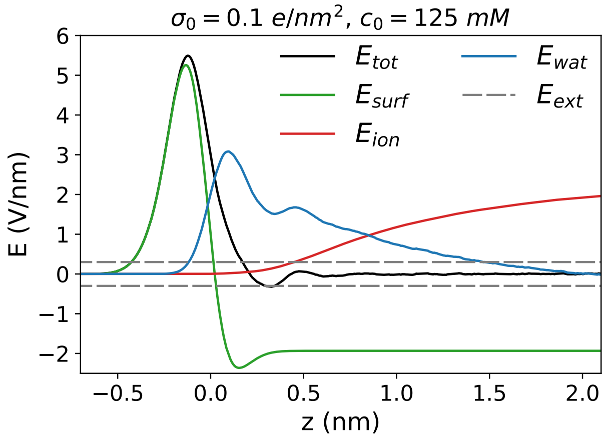


FIG. 1. Magnitude of the simulated electric field $E(z)$ (black solid line, denoted $E_{\rm tot}$), split into the contributions from the surface (green solid line), the ions (red solid line) and the water (blue solid line). The applied electric field (black dashed lines) in the nonequilibrium electrokinetic simulations is shown for comparison.

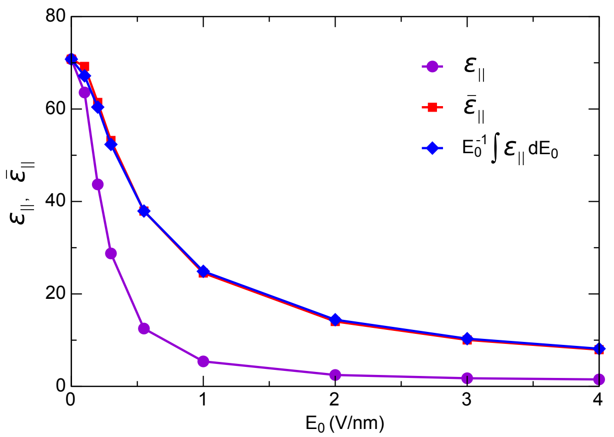


FIG. 2. The dielectric differential constant $\varepsilon_{\|}(E_0, 0)$ calculated from the fluctuation equation, the dielectric difference constant $\bar{\varepsilon}_{\|}(E, 0)$, and the integral of Eq. (4). All calculated at zero concentration, $c_0 = 0$.

## I. LOCAL ELECTRIC FIELD

In the simulations, the electric field perpendicular to the surface is given by the integral over all charges,

$$E(z) = \int_{-\infty}^{z} dz' \left(\rho_{\rm ion}(z') + \rho_{\rm surf}(z') + \rho_{\rm wat}(z')\right), \qquad (1)$$

with $\rho_{\rm ion}(z)$ being the ionic charge density, $\rho_{\rm surf}(z)$ being the density of charges on the surface, and $\rho_{\rm wat}(z)$ being the density of partial charges on the water molecules. In practice, we use the edge of the simulation box for the lower limit of the integral. In Fig. 1, we show the field $E(z) = E_{\rm ion}(z) + E_{\rm surf}(z) + E_{\rm wat}(z)$, together with the electric field applied parallel to the surface in the nonequilibrium simulations, $E_{\rm ext}$. Clearly, each of the contributions exceeds the external electric field, justifying our approximation to consider only the field perpendicular to the surface.

* These authors contributed equally.
† bonthuis@tugraz.at

## II. DEFINITION OF THE DIELECTRIC CONSTANT

The local dielectric constant is defined as the change of displacement field in response to a small change in electric field,

$$dD(z) = \varepsilon_{\|}(E(z), c(z), z)\varepsilon_0 dE(z). \qquad (2)$$

To calculate the total displacement field in response to an electric field, we formulate the Poisson equation as

$$D(z) = \bar{\varepsilon}_{\|}(E(z), c(z), z)\varepsilon_0 E(z). \qquad (3)$$

Because of the electric-field dependence of the dielectric constant, the dielectric difference constant $\bar{\varepsilon}_{\|}(E(z), c(z), z)$ does not equal the dielectric differential constant $\varepsilon_{\|}(E(z), c(z), z)$. In the bulk, the two are related by

$$\bar{\varepsilon}_{\|}(E_0, c_0) = \frac{1}{E_0}\int_0^{E_0} \varepsilon_{\|}(E_0', c_0) dE_0'. \qquad (4)$$

In Fig. 2, we show the results from the simulations of $\varepsilon_{\|}(E_0, c_0)$, $\bar{\varepsilon}_{\|}(E_0, c_0)$ and Eq. (4), showing perfect agreement between the latter two.

## III. CALCULATION OF THE VELOCITY PROFILE

In the $z$ direction (perpendicular to the surface), the simulation box is cut into slabs of width $dz$. The velocity profile in the non-equilibrium electrokinetic simulations is calculated by averaging the velocity of all atoms in a slab between $z$ and $z + dz$. To find the velocity profiles in Fig. 5 of the main text, the average velocity of the atoms belonging to the surface is subtracted.
